# Relative Aerobic Load of Daily Activities After Stroke

**DOI:** 10.1093/ptj/pzad005

**Published:** 2023-01-16

**Authors:** Ilse J Blokland, Linda F A Schiphorst, Jessie R Stroek, Floor P Groot, Coen A M van Bennekom, Jaap H van Dieen, Jos J de Koning, Han Houdijk

**Affiliations:** Department of Human Movement Sciences, Faculty of Behavioural and Movement Sciences, Vrije Universiteit Amsterdam, Amsterdam Movement Sciences, Amsterdam, The Netherlands; Heliomare Research and Development, Heliomare, Wijkaan Zee, The Netherlands; Department of Human Movement Sciences, Faculty of Behavioural and Movement Sciences, Vrije Universiteit Amsterdam, Amsterdam Movement Sciences, Amsterdam, The Netherlands; Heliomare Research and Development, Heliomare, Wijkaan Zee, The Netherlands; Department of Human Movement Sciences, Faculty of Behavioural and Movement Sciences, Vrije Universiteit Amsterdam, Amsterdam Movement Sciences, Amsterdam, The Netherlands; Heliomare Research and Development, Heliomare, Wijkaan Zee, The Netherlands; Sport- en BeweegKliniek, Haarlem, The Netherlands; Coronel Institute of Occupational Health, Amsterdam University Medical Center, University of Amsterdam, Amsterdam, The Netherlands; Department of Human Movement Sciences, Faculty of Behavioural and Movement Sciences, Vrije Universiteit Amsterdam, Amsterdam Movement Sciences, Amsterdam, The Netherlands; Department of Human Movement Sciences, Faculty of Behavioural and Movement Sciences, Vrije Universiteit Amsterdam, Amsterdam Movement Sciences, Amsterdam, The Netherlands; University of Groningen, University Medical Center Groningen, Center for Human Movement Sciences, Groningen, The Netherlands

**Keywords:** Activities of Daily Living, Energy Cost, Energy Expenditure, Gait, Gait Disorders: Neurologic

## Abstract

**Objective:**

Individuals after stroke are less active, experience more fatigue, and perform activities at a slower pace than peers with no impairments. These problems might be caused by an increased aerobic energy expenditure during daily tasks and a decreased aerobic capacity after stroke. The aim of this study was to quantify relative aerobic load (ie, the ratio between aerobic energy expenditure and aerobic capacity) during daily-life activities after stroke.

**Methods:**

Seventy-nine individuals after stroke (14 in Functional Ambulation Category [FAC] 3, 25 in FAC 4, and 40 in FAC 5) and 22 peers matched for age, sex, and body mass index performed a maximal exercise test and 5 daily-life activities at a preferred pace for 5 minutes. Aerobic energy expenditure (mL O_2_/kg/min) and economy (mL O_2_/kg/unit of distance) were derived from oxygen uptake ($\dot{\mathrm{V}}{\mathrm{O}}_2$). Relative aerobic load was defined as aerobic energy expenditure divided by peak aerobic capacity (%$\dot{\mathrm{V}}{\mathrm{O}}_2$peak) and by $\dot{\mathrm{V}}{\mathrm{o}}_2$ at the ventilatory threshold (%$\dot{\mathrm{V}}{\mathrm{o}}_2$-VT) and compared in individuals after stroke and individuals with no impairments.

**Results:**

Individuals after stroke performed activities at a significantly higher relative aerobic load (39%–82% $\dot{\mathrm{V}}{\mathrm{o}}_2$peak) than peers with no impairments (38%–66% $\dot{\mathrm{V}}{\mathrm{o}}_2$peak), despite moving at a significantly slower pace. Aerobic capacity in individuals after stroke was significantly lower than that in peers with no impairments. Movement was less economical in individuals after stroke than in peers with no impairments.

**Conclusion:**

Individuals after stroke experience a high relative aerobic load during cyclic daily-life activities, despite adopting a slower movement pace than peers with no impairments. Perhaps individuals after stroke limit their movement pace to operate at sustainable relative aerobic load levels at the expense of pace and economy.

**Impact:**

Improving aerobic capacity through structured aerobic training in a rehabilitation program should be further investigated as a potential intervention to improve mobility and functioning after stroke.

## Introduction

Individuals after stroke are less active, experience more fatigue, and perform activities at a slower pace compared with individuals with no impairments.^1^^,^^2^ This can compromise their daily-life participation. In part, these mobility problems can be explained by underlying neuromuscular impairments such as reduced motor control, muscle weakness, and spasticity. But an additional reason might be their reduced aerobic capacity combined with an increased aerobic energy expenditure during daily activities after stroke.

The physiological demand of a task is dependent not only on the absolute aerobic energy requirement of the task but also on the percentage of the individual’s aerobic capacity that is engaged. The physiological demand can be described by the ratio between aerobic energy expenditure and the available aerobic capacity: the relative aerobic load. At an exercise intensity above 40% to 60% of maximal aerobic capacity, anaerobic energy pathways are increasingly used, which results in increased lactate turnover, changes in respiration, and fatigue; this intensity is referred to as the ventilatory threshold (VT). Above the VT, exercise cannot be sustained for a prolonged period of time.^3^ Thus, relative aerobic load—and not absolute aerobic energy requirement—determines the strain experienced, the level of fatigue that will occur during the task, and the time this task can be sustained.^4^^,^^5^

A systematic review of 41 studies reported that the aerobic energy capacity of individuals after stroke was, on average, 53% that of individuals with no impairments.^6^ On top of that, movement economy (ie, the aerobic energy expenditure normalized to pace; energy required per unit distance) is deteriorated after stroke, and aerobic energy expenditure during various activities has been found to be higher in individuals after stroke compared with peers with no impairments.^2^^,^^7–9^ Given the lower aerobic capacity and increased energy expenditure, relative aerobic load will likely also be higher in individuals after stroke.

An effective strategy to reduce relative aerobic load is lowering movement pace. Lowering walking pace to reach sustainable levels of relative aerobic load has previously been observed in people after stroke^9^ and in other patient populations (eg, people after lower-limb amputation^10^). Reducing relative aerobic load at the expense of pace will allow individuals to perform activities for a longer duration, limit fatigue development, or to achieve a combination of both. Hence, a high relative aerobic load could be a cause of the lower movement pace in individuals after stroke.

Studies on aerobic load after stroke have mostly focused on absolute aerobic energy expenditure without including measures of aerobic capacity and were mostly limited to the task of walking.^4^^,^^11^ Relative aerobic load of walking after stroke has been assessed in only a few studies. Relative aerobic loads of 62% to 65% peak aerobic capacity ($\dot{\mathrm{V}}{\mathrm{o}}_2$peak) during walking have been reported for individuals after stroke walking at preferred speeds of 0.62 and 0.89 m/s, respectively.^12^^,^^13^ However, these studies lacked comparisons with peers with no impairments. Recently, we directly compared relative aerobic load of treadmill walking between individuals with no impairments and individuals after stroke at preferred walking speed. We found an average relative aerobic load of 50% $\dot{\mathrm{V}}{\mathrm{o}}_2$peak for individuals after stroke, compared with 36% $\dot{\mathrm{V}}{\mathrm{o}}_2$peak for individuals with no impairments even though they walked significantly faster.^9^ However, it is unclear how these findings translate to other functional activities.

The absolute aerobic load after stroke during other functional activities such as stair ambulation and washing dishes was reported to be higher than available compendium values for individuals with no impairments,^7^ but this study did not include measures of aerobic capacity*.* So, the consequences of the increased absolute aerobic load in terms of relative aerobic load cannot be derived from this study, and potential requirements for training aerobic fitness to cope with this increased relative load are overlooked. Only 2 studies quantified the relative aerobic load of activities other than walking: Serra et al^14^ reported relative aerobic loads between 17% and 65% $\dot{\mathrm{V}}{\mathrm{o}}_2$peak in different tasks such as TV watching and stepping in place. Unfortunately, pace was not reported in this study for the activities other than walking. Gjellesvik et al^13^ found a relative aerobic load of 61% $\dot{\mathrm{V}}{\mathrm{o}}_2$peak in the final 30 seconds of a field test containing 5 functional activities. However, in these studies, the relative aerobic load was only presented in a secondary analysis and neither study included a control group. Thus, a direct comparison of the relative aerobic load of different functional activities with peers with no impairments is lacking. Also, the effect of stroke severity on relative aerobic load is unknown because individuals after stroke were not stratified by functional level in these studies.

The aim of this study was to quantify the level of relative aerobic load of daily-life activities in individuals after stroke compared with individuals with no impairments. In addition, movement pace and economy of these activities were compared between groups. To investigate the effect of stroke severity on our outcome measures, the individuals after stroke were stratified by Functional Ambulation Category (FAC).^15^ Based on previous results in walking,^9^ we hypothesized that individuals after stroke would experience a higher relative aerobic load during daily activities, coinciding with lower movement pace, lower movement economy, and lower aerobic capacity. We expected these effects to be more pronounced in individuals with lower levels of functional ambulation. To control for the effect of movement speed on relative aerobic load and economy we also compared the relative aerobic load of individuals after stroke with that of individuals with no impairments moving at a speed comparable to that of individuals after stroke. Based on this study, recommendations can be given regarding the importance of exercise training to reduce relative aerobic load, improve movement pace, reduce fatigue, and consequently enhance functioning in individuals after stroke.

## Methods

Eighty-six individuals in the subacute phase after stroke and 22 individuals with no impairments were enrolled in this cross-sectional study. Individuals after stroke were all inpatients in the rehabilitation center and were referred for a routine cardiopulmonary exercise test. Seventy-nine individuals after stroke completed the protocol and were included in the analysis.

Inclusion criteria for individuals after stroke were older than 17 years of age and FAC greater than 2, indicating the ability to walk without manual assistance. Exclusion criteria for all participants were contraindications for cardiopulmonary exercise testing and/or exercise^16^^,^^17^ and the inability to understand or perform study instructions. Additionally, individuals with no impairments were excluded if they had a history of stroke or heart disease or if they had a disorder influencing walking ability. The participants with no impairments were matched at group level to the age, sex, and body mass index of individuals after stroke. This study was approved by the Medical Ethical Committee of the VU Medical Center (NL-64431.029.18). All participants were fully informed about the study aim and protocol and signed written informed consent before participation.

Because previous studies showed that the speed and aerobic energy expenditure of walking in individuals after stroke depend on the level of impairment,^9^^,^^18^ individuals after stroke were stratified based on FAC: FAC 3, representing independent walking ability under supervision; FAC 4, representing ability to walk on level terrain without supervision; and FAC 5, representing independent walking and stair ambulation ability.

### Procedures

Participants performed a cardiopulmonary exercise test and an activity protocol consisting of five 5-minute activity trials scheduled 2 to 14 days apart. Participants were asked to refrain from heavy exercise 24 hours before the measurements and to refrain from taking food and caffeinated beverages 2 hours before the measurements. Before testing, participants’ body height and mass were measured. Additionally, for individuals after stroke, the Motricity Index^19^ and the FAC^15^ were determined. The following characteristics were retrieved from patient files: time since stroke, side and type of stroke, first or recurrent stroke, and days since admission.

#### Cardiopulmonary Exercise Test

The cardiopulmonary exercise test was performed on an electronically braked cycle ergometer under the supervision of an experienced physician. After a 3-minute rest phase and a 3-minute warm-up at 0 W and 50 to 70 revolutions per minute, a ramp protocol based on the estimated maximum exercise capacity of the participant was used, so that the exercise phase was expected to be about 8 to 12 minutes. The ramp phase was stopped if the participant could not sustain a pace of 50 revolutions per minute, if the physician deemed it unwise to continue the test, or if the participant wanted to stop. A cool-down phase of 3 minutes at 10% of peak power followed.

Breath-by-breath gas exchange (Jaeger Oxycon Pro; Vyaire, Hoechberg, Germany) and arterial oxygen saturation were continuously measured. Additionally, for individuals after stroke, a 12-lead electrocardiogram and blood pressure were monitored.

#### Activity Protocol

Participants performed the following 5 activities continuously for 5 minutes: walking back and forth in a 30-m level corridor; walking an obstacle course back and forth in the same corridor (Suppl. Appendix SI); ambulation on a staircase with 8 steps; sweeping leaves from a 9-m^2^ field (Suppl. Appendix SI); and cycling on an ergometer. These activities were chosen with an experienced physical therapist and an experienced occupational therapist to ensure these were relevant and common targets of practice in rehabilitation of people after stroke. Activities were performed in a randomized order at the preferred movement pace (or preferred power for cycling) of the participant. Additionally, participants with no impairments repeated each activity at half their preferred pace, which was expected to be about the average self-selected pace of the individuals after stroke. The pace for this trial was verbally indicated by the researcher. Seated rest between trials was at least 5 minutes to ensure a return to resting metabolic values. Breath-by-breath gas exchange and heart rate (Polar Wearlink Strap; Polar, Kempele, Finland) were monitored continuously during the trials.

### Data Analysis

Peak aerobic capacity ($\dot{\mathrm{V}}{\mathrm{o}}_2$peak; ie, peak rate of oxygen uptake) was determined from the cardiopulmonary exercise test data. The maximum of the 30-second averaged oxygen uptake was considered $\dot{\mathrm{V}}{\mathrm{o}}_2$peak. $\dot{\mathrm{V}}{\mathrm{o}}_2$peak was considered valid when a participant reached a respiratory quotient exceeding 1.1 (>1.0 for participants over 65 years of age^20^).

Oxygen uptake at VT ($\dot{\mathrm{V}}{\mathrm{o}}_2$-VT) was used as an additional measure of aerobic capacity. Exercise intensities below $\dot{\mathrm{V}}{\mathrm{o}}_2$-VT can be sustained for a long duration without involvement of anaerobic metabolism, and $\dot{\mathrm{V}}{\mathrm{o}}_2$-VT has been suggested to be a more specific measure of endurance for individuals after stroke.^21^ Two assessors determined VT following the V-slope method.^22^ A plot of the ventilatory equivalents and respiratory quotient over time was presented after V-slope assessment to allow the assessor to adjust the time point of VT if needed. If the 2 assessors did not agree on the $\dot{\mathrm{V}}{\mathrm{o}}_2$-VT within 100 mL O_2_/min, then they conferred to reach a consensus. This occurred in 10 of 101 cases.

For the activity trials, aerobic energy expenditure (in mL O_2_/kg/min) was determined by averaging $\dot{\mathrm{V}}{\mathrm{O}}_2$ during the last 2 minutes of each trial. Relative aerobic load was defined as aerobic energy expenditure divided by $\dot{\mathrm{V}}{\mathrm{O}}_2$peak (%$\dot{\mathrm{V}}{\mathrm{o}}_2$peak) and $\dot{\mathrm{V}}{\mathrm{o}}_2$-VT (%$\dot{\mathrm{V}}{\mathrm{o}}_2$-VT). Pace was defined as m/s for walking, steps per second for stair ambulation, and m^2^/s for sweeping. For cycling, movement intensity was expressed not as pace but as mechanical power, in watts (J/s), whereas pedaling rate was kept constant at between 50 and 70 revolutions per minute. Economy was calculated by dividing aerobic energy expenditure by pace; thus, lower values indicate better movement economy.

### Statistical Analysis

Data were analyzed using SPSS (IBM SPSS Statistics version 25.0; IBM Corp, Chicago, IL, USA) and checked for normality using visual inspection, skewness, and kurtosis. A *P* value <.05 was considered significant.

Because multiple variables did not meet the criteria for normality, the analysis was performed with nonparametric tests. We performed Kruskal-Wallis χ^2^ tests for main effects of group on pace, aerobic energy expenditure, economy, and relative aerobic load (in both %$\dot{\mathrm{V}}{\mathrm{o}}_2$-VT and %$\dot{\mathrm{V}}{\mathrm{o}}_2$peak) for each separate activity. Post hoc testing was limited a priori to testing for differences between the control group and either of the subgroups of individuals after stroke stratified for FAC score, using Mann-Whitney *U* tests with Bonferroni correction per activity [*P* < (.05/5 = .01)]. All analyses were performed twice: once with the control group at the preferred pace and once with the control group at half the preferred pace.

## Results

### Participants

Seventy-nine of 86 individuals after stroke were included in the study: 14 individuals in FAC 3, 25 in FAC 4, and 40 in FAC 5. Seven individuals after stroke performed only the cardiopulmonary exercise test and did not perform any activity trials. Four found the protocol too tiring in combination with rehabilitation, 1 found the breathing mask uncomfortable, 1 was discharged early, and 1 experienced an attack of gout before the planned measurement. All 22 participants with no impairments completed the protocol. Not all participants started or completed every activity, and some trials were excluded from analysis (Suppl. Appendix SII). For each group, 73% to 99% of potential trials were included in the analysis.

Group characteristics are displayed in the Table. No significant differences existed for age and sex. Although we matched the control group for body mass index at the group level with the complete group of individuals after stroke, the body mass index of the FAC 5 group was significantly higher than that of the other groups.

#### Aerobic Capacity

Both $\dot{\mathrm{V}}{\mathrm{o}}_2$peak and $\dot{\mathrm{V}}{\mathrm{o}}_2$-VT of the individuals after stroke were significantly lower (on average, 54%–63%) than those of the group with no impairments (Table).

**Table:** Group Characteristics*^a^*

**Characteristic**	**FAC 3 (n = 14)**	**FAC 4 (n = 25)**	**FAC 5 (n = 40)**	**No Impairments (n = 22)**
Age, y, mean (SD)	60.3 (14.2)	56.0 (12.2)	60.9 (11.3)	60.1 (6.3)
Sex, men/women (% men)	12/2 (86)	16/9 (64)	32/8 (80)	14/4 (78)
BMI, kg/m^2^, mean (SD)	24.1 (2.4)*^b^*	25.5 (4.3)*^b^*	26.6 (3.8)	24.2 (3.6)*^b^*
$\dot{\mathrm{V}}{\mathrm{o}}_2$ peak, mL/kg/min, mean (SD)	18.4 (5.9) (n = 13)*^c^*	19.5 (6.1) (n = 23)*^c^*	21.0 (6.0) (n = 39)*^c^*	33.4 (7.7)
$\dot{\mathrm{V}}{\mathrm{o}}_2$ -VT, mL/kg/min, mean (SD)	9.7 (2.4)*^c^*	9.9 (2.7)*^c^*	10.9 (2.8)*^c^*	18.1 (5.7)
Time since stroke, d, median (range)	48.5 (58)	28 (28)	20 (28)	
Time since admission, d, median (range)	23.5 (26)	15 (24)	13 (21)	
Side of stroke, left/right/other, no. of participants	7/6/1	10/10/5	21/14/5	
Stroke type, ischemic/hemorrhagic, no. of participants	12/2	19/6	31/9	
First/recurrent stroke, no. of participants	11/3	21/4	34/6	
Use of walking aid in daily life, yes/no, no. of participants	12/2	14/11	8/32	
Beta-blocker medication, yes/no, no. of participants	3/11	7/18	11/29	
Motricity Index, mean (SD)	49.6 (25.6)	72.8 (21.9)*^d^*	84.5 (13.8)*^d^*^,^*^e^*	
Lower extremity	53.4 (27.8)	76.0 (22.3)*^d^*	86.8 (13.5)*^d^*	
Upper extremity	45.8 (33.7)	69.5 (25.8)*^d^*	82.2 (18.6)*^d^*	
Trials completed (% of total)	51 (72.6)	115 (92)	187 (94)	218 (99.1)
Walking	14	25	38	44
Obstacles	8	22	38	44
Sweeping	5	23	38	44
Stairs	10	23	34	42
Cycling	14	22	39	44

^a^ Values are presented as number (percentage) of participants unless otherwise indicated. BMI = body mass index; FAC = Functional Ambulation Category; $\dot{\mathrm{V}}{\mathrm{o}}_2$peak = peak aerobic capacity; $\dot{\mathrm{V}}{\mathrm{o}}_2$-VT = oxygen uptake at the ventilatory threshold.

^b^ Significantly different from FAC 5.

^c^ Significantly different from group with no impairments.

^d^ Significantly different from FAC 3.

^e^ Significantly different from FAC 4.

#### Pace

**Figure 1:**
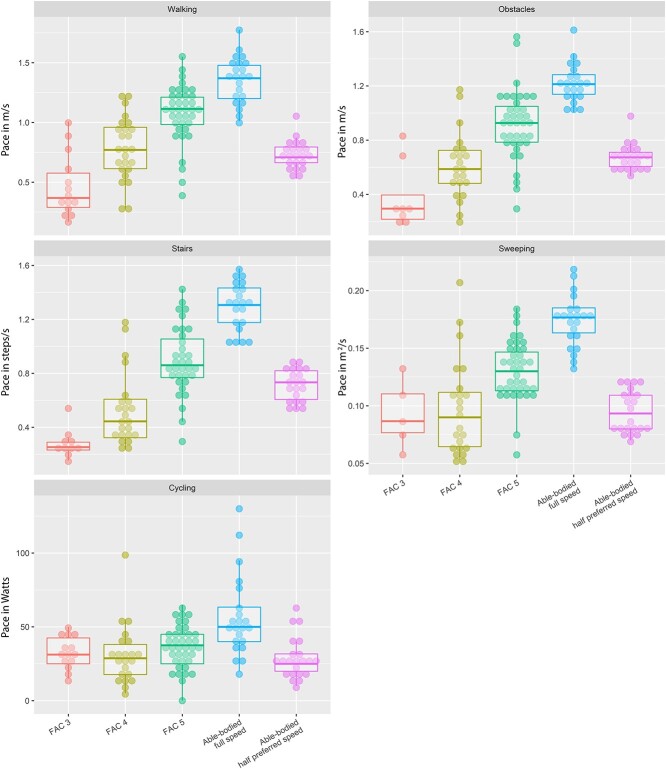
Movement pace displayed per subgroup for each activity. Pace was defined as m/s for walking, steps/s for stair ambulation, and m^2^/s for sweeping. For cycling, movement intensity was expressed not as pace but as mechanical power, in watts (J/s). Dots represent individual participants. Individuals with no impairments at full pace were similar to those with no impairments at half the preferred pace.

Pace was significantly different between groups during all activities (*P* < .000). In general, Figure 1 shows that average pace was highest in the group with no impairments and decreased with decreasing FAC score. Post hoc tests showed that the group with no impairments moved significantly faster, during all activities, than all subgroups of individuals after stroke (*P* < .002).

When the results at half the preferred pace for the group with no impairments were considered, pace was still significantly different between groups during all activities (*P* < .000) except cycling (*P* = .075). Post hoc tests showed that the FAC 3 group moved significantly more slowly than the control group at half the preferred pace during walking, walking with obstacles, and stair climbing. The FAC 4 group moved significantly more slowly than individuals with no impairments at half the preferred pace during stair climbing. The FAC 5 group moved significantly more quickly than the control group at half the preferred pace during all activities.

#### Aerobic Energy Expenditure

**Figure 2:**
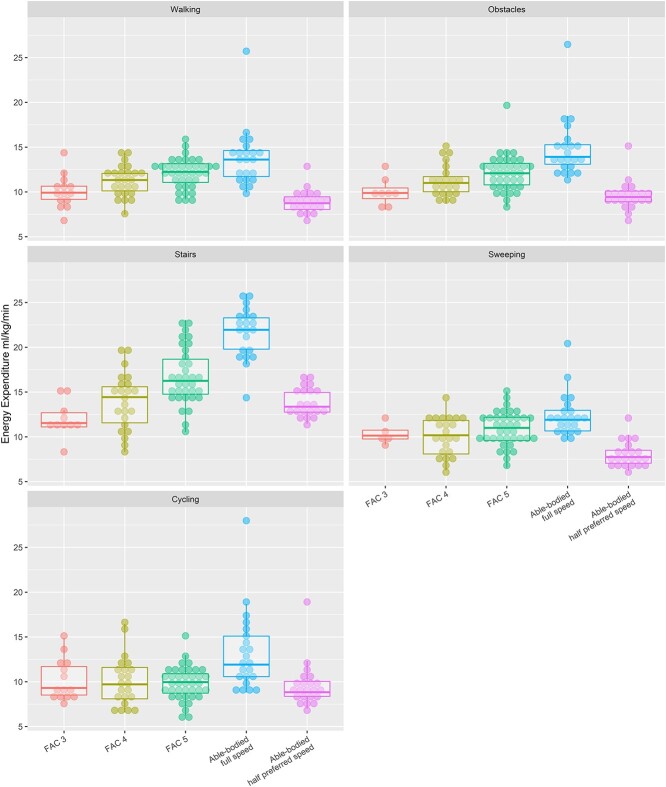
Aerobic energy expenditure expressed as mL O_2_/kg/min and displayed per subgroup for each activity. Dots represent individual participants. Individuals with no impairments at full pace were similar to those with no impairments at half the preferred pace.

Absolute aerobic energy expenditure was significantly different between groups during all activities (*P* < .016). In general, Figure 2 shows that average aerobic energy expenditure was highest in the group with no impairments and decreased with decreasing FAC score. However, this pattern was less clear for sweeping and cycling. Post hoc tests showed either that aerobic energy expenditure in all of the FAC groups was significantly lower than that in the group with no impairments or that this difference approached significance (FAC 3 for sweeping [*P* = .047] and cycling [*P* = .014]; FAC 5 for walking [*P* = .022] and sweeping [*P* = .029]).

When the results at half the preferred pace for the group with no impairments were considered, aerobic energy expenditure was still significantly different between groups for all activities except cycling. Post hoc tests showed that aerobic energy expenditure during sweeping in all FAC groups was significantly higher than that in the group with no impairments at half the preferred pace. Furthermore, aerobic energy expenditure during walking and walking with obstacles in the FAC 4 and FAC 5 groups was higher than that in the group with no impairments at half the preferred pace, whereas aerobic energy expenditure of stair ambulation was significantly lower in the FAC 3 group than in the group with no impairments at half the preferred pace. Aerobic energy expenditure was significantly higher in the FAC 5 group than in the group with no impairments at half the preferred pace.

#### Relative Aerobic Load

**Figure 3:**
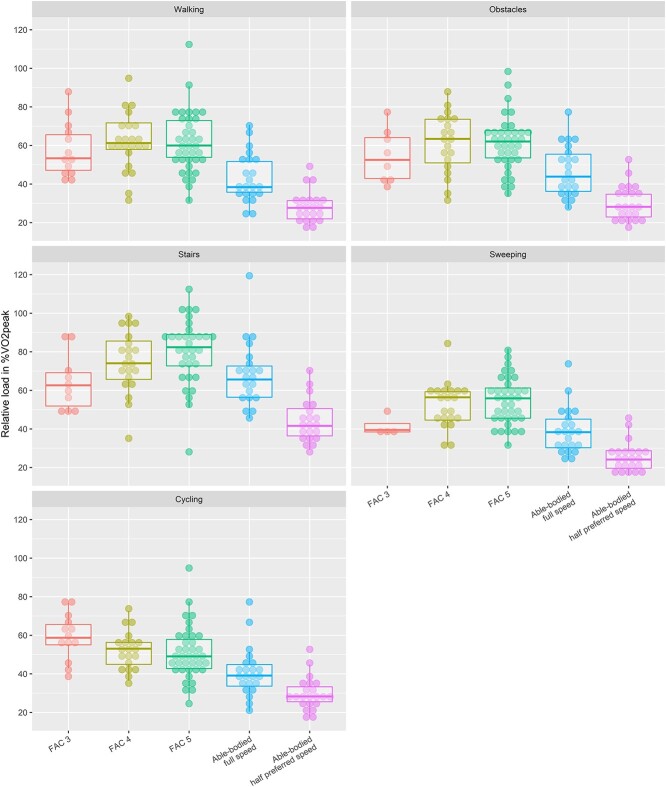
Relative aerobic load expressed as aerobic energy expenditure divided by peak aerobic capacity (%$\dot{\mathrm{V}}{\mathrm{o}}_2$peak) and displayed per subgroup for each activity. Dots represent individual participants. Individuals with no impairments at full pace were similar to those with no impairments at half the preferred pace. Some participants were excluded from this analysis because they did not reach the criteria for maximal effort (Table).

**Figure 4:**
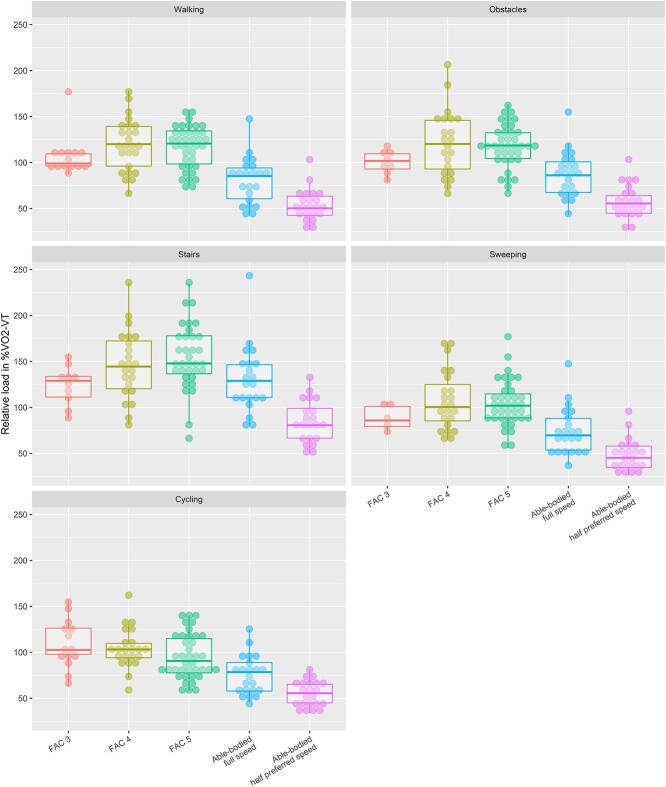
Relative aerobic load expressed as aerobic energy expenditure divided by oxygen uptake at the ventilatory threshold (%$\dot{\mathrm{V}}{\mathrm{o}}_2$-VT) and displayed per subgroup for each activity. Dots represent individual participants. Individuals with no impairments at full pace were similar to those with no impairments at half the preferred pace.

Relative aerobic load (expressed as %$\dot{\mathrm{V}}{\mathrm{o}}_2$peak and $\%\dot{\mathrm{V}}{\mathrm{o}}_2$-VT) was significantly different between groups during all activities (*P* < .004) (Figs. 3 and 4). Post hoc testing showed that %$\dot{\mathrm{V}}{\mathrm{o}}_2$peak in the control group was lower than that in the FAC 4 and FAC 5 groups during all activities and that it was also lower than that in the FAC 3 group during walking and cycling. Differences in relative aerobic load were even more pronounced when the results for the group with no impairments at half the preferred pace were considered. In this comparison, %$\dot{\mathrm{V}}{\mathrm{o}}_2$peak was higher in the group with no impairments than in all stroke subgroups during all activities (Fig. 3). Effects on relative aerobic load in %$\dot{\mathrm{V}}{\mathrm{o}}_2$-VT were similar to those on %$\dot{\mathrm{V}}{\mathrm{o}}_2$peak (Fig. 4).

#### Economy

**Figure 5:**
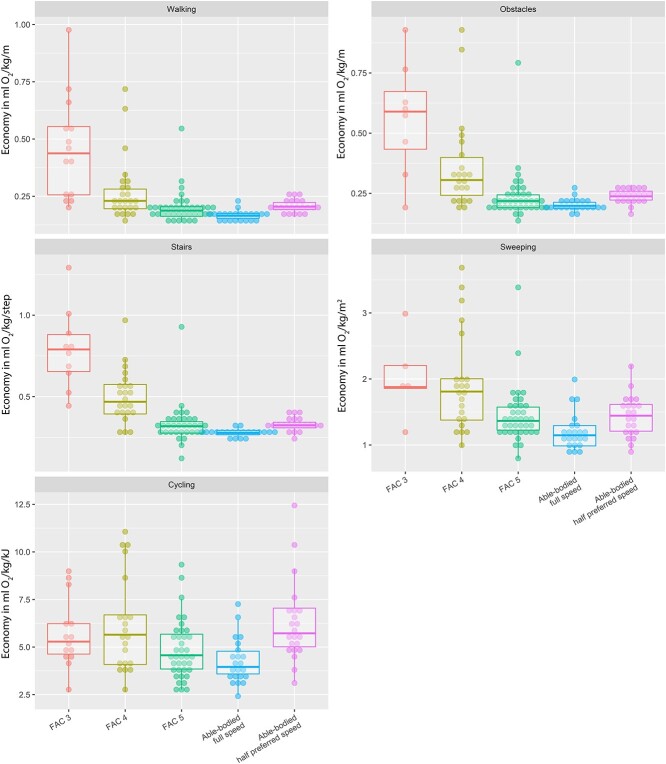
Economy expressed as mL O_2_/kg/unit of distance or mL O_2_/kg/kJ and displayed per subgroup for each activity. Individuals with no impairments at full pace were similar to those with no impairments at half the preferred pace.

Economy was significantly different between groups during all activities (*P* < .005) (Fig. 5). Post hoc testing showed that economy in the group with no impairments was significantly better than economy in the FAC 3 and FAC 4 groups during all activities. Economy in the group with no impairments was only significantly better than that in the FAC 5 group during sweeping and stair ambulation, and this difference approached significance during walking with and without obstacles (*P* = .018 and *P* = .046, respectively).

When the results for the group with no impairments at half the preferred pace were considered, economy was also significantly different between groups for all activities (*P* < .000). Post hoc tests showed that economy in the group with no impairments at half the preferred pace was better than that in the FAC 3 and FAC 4 groups and comparable to that in the FAC 5 group for all activities except cycling. Cycling economy in the control group at half the preferred pace was comparable to that in the FAC 3 and FAC 4 groups and worse than that in the FAC 5 group.

## Discussion

This study compared the relative aerobic load of cyclic daily-life activities in individuals after stroke with that of peers with no impairments*.* In line with our hypothesis, we found that individuals after stroke performed activities at a significantly higher relative aerobic load than peers with no impairments, despite moving at a significantly slower pace. Also in line with our hypotheses, both aerobic capacity and movement economy were worse after stroke. These findings indicate that, in addition to neuromuscular impairments, physiological limitations might cause mobility limitations in individuals after stroke.

Due to the decrease in movement pace, the aerobic energy expenditure of individuals after stroke was equal to or lower than that of individuals with no impairments. Nevertheless, this resulted in a higher relative aerobic load as the aerobic capacity of individuals after stroke was about 60% that of individuals with no impairments in our sample (Table). We found average relative aerobic loads of the different activities between 39% $\dot{\mathrm{V}}{\mathrm{o}}_2$peak (sweeping in the FAC 3 group) and 82% $\dot{\mathrm{V}}{\mathrm{o}}_2$peak (stair ambulation in the FAC 5 group) in individuals after stroke compared with 38% to 66% $\dot{\mathrm{V}}{\mathrm{o}}_2$peak in individuals with no impairments. The difference in relative aerobic load was even more pronounced when individuals with no impairments performed activities at half preferred pace, which was comparable to the average pace of the individuals after stroke (24%–42% $\dot{\mathrm{V}}{\mathrm{o}}_2$peak).

The relative aerobic load of walking at preferred speed found in our stroke population (55%–61% $\dot{\mathrm{V}}{\mathrm{o}}_2$peak) was comparable to that reported in other studies on individuals after stroke^9^^,^^12^^,^^13^ and individuals with other disorders such as lower-limb amputation, chronic heart failure, chronic obstructive pulmonary disease, and coronary artery disease.^10^^,^^23–25^ Interestingly, relative aerobic load values in all these studies were all close to the VT for sustained, self-paced activities, which occurs at about 40% to 60% $\dot{\mathrm{V}}{\mathrm{o}}_2$peak in individuals who are not trained.^3^ This was also true for other self-paced activities in our study and previous studies.^13^^,^^14^ In 2 previous studies, relative aerobic load only exceeded standard VT values when movement pace was set above preferred speed.^9^^,^^14^ Our study showed that in contrast, individuals with no impairments stayed well below the VT during all activities except during stair ambulation. This indicates that for a majority of individuals after stroke, increasing pace towards that of peers with no impairments would result in an unsustainable relative aerobic load and early fatigue, as we have shown previously in walking.^9^ Based on these findings one could speculate that individuals after stroke limit their pace, to limit relative aerobic load levels to such an extent that they are able to sustain the activity.

Participants in FAC 3 are likely not primarily limited in movement speed by a high relative aerobic load. They tended to move at the lowest pace but also at a lower relative aerobic load than participants in FAC 4 and FAC 5. Hence, for individuals after stroke in FAC 3, neuromuscular control limitations might be a more important determinant of reduced movement pace than relative aerobic load levels.

Although a lower movement pace has the advantage of lowering relative aerobic load, the downside is that it may lead to a decrease in economy. Because economy is parabolically related with walking speed, lowering walking speed generally leads to a decrease in walking economy as individuals move further away from their most economic speed.^9^^,^^18^^,^^26^ This phenomenon was observed in all activities of our participants with no impairments when they were instructed to move at half preferred pace, and in our previous study when individuals after stroke walked below their preferred speed on a treadmill^9^; their aerobic energy expenditure and relative aerobic load clearly decreased, but at the expense of movement economy. Thus, when individuals after stroke would be able to sustain a higher movement pace, this might result in a more economic movement pattern.

The difference between individuals with no impairments and individuals after stroke observed for cycling tended to differ from the other activities. Individuals with higher functional ambulation showed a higher pace, higher absolute load and better economy during walking, obstacle walking, and stair ambulation compared with individuals in a lower FAC. In these tasks, average relative aerobic load was highest in the FAC 4 and FAC 5 groups. In cycling, however, pace appeared comparable between stroke subgroups and relative load was on average highest in the FAC 3 group. During cycling, body mass is supported by the bicycle seat and performing this task on an ergometer does not require balance control. Thus, during cycling, movement pace might be less limited by impairments in neuromuscular control. Consequently, during this activity more impaired individuals may limit their pace based on relative aerobic load instead of neuromuscular limitation.

The results of this study have important consequences for the rehabilitation program for people after stroke. Aside from a focus on neuromuscular control and functional ambulation training as advised by the American Heart Association,^27^ structured aerobic training should be further investigated and considered in rehabilitation after stroke to improve mobility—not only to reduce the risk of comorbidities and recurring stroke, but also to reach a direct functional gain in mobility. For example, if a patient seems to be limited by movement speed or fatigue, a measure of aerobic capacity combined with a measure of aerobic load of walking could reveal potential underlying aerobic limitations and further inform treatment decisions. Exercise training might enhance aerobic capacity, reduce relative aerobic load, improve movement pace, reduce fatigue, and enhance movement economy. Clinical studies on these specific effects of fitness training on individuals after stroke are still scarce in literature. However, Macko et al^12^ previously showed that after an aerobic treadmill training program for individuals after stroke, the relative aerobic load of walking decreased from 62% to 50% $\dot{\mathrm{V}}{\mathrm{o}}_2$peak, whereas walking speed increased. These data indicate that individuals not only reduced relative aerobic load but also exploited the training effect to increase movement speed. It cannot be determined to what extent an improved economy or an improved aerobic capacity contributed to this reduced relative load and increased speed. Nevertheless, it seems likely that improving aerobic capacity would make it possible to sustain a higher walking speed, which would in turn lead to a better walking economy. We hope that our study’s results on these underlying mechanisms further motivate the implementation of aerobic training in rehabilitation programs of individuals after stroke and longitudinal research into clinical effects.

### Limitations

Some limitations should be considered when interpreting the results of this study. First, variation in performance among individuals after stroke in separate FAC groups is known to be large.^15^ Therefore, statistical differences between FAC groups are not easily detected and we only focused on difference between FAC groups and the group with no impairments. Although the observed trends regarding the effect of FAC are consistent between tasks and parameters, the effect of FAC needs to be interpreted with caution. The FAC 3 group was small compared with the other groups because fewer individuals referred for a cardiopulmonary exercise test during inclusion belonged to this group. Furthermore, in this group, not all individuals could perform all activities. This likely led to a selection of relatively high-functioning individuals in the FAC 3 group. In sweeping, for example, we found comparable values of pace and aerobic energy expenditure for the FAC 3 and FAC 4 groups. However, 9 of 14 individuals in the FAC 3 group could not perform this task due to functional limitations. Still, our results seem to indicate that this subgrouping is relevant as the FAC 3 group seemed to behave differently in several tasks.

Furthermore, the difference in relative aerobic load between individuals with no impairments and individuals after stroke might be aggravated by the fact that the participants with no impairments in this study participated voluntarily in a cardiopulmonary exercise test, which may have attracted relatively fit individuals. On the other hand, individuals after stroke in this study arguably also were a more fit selection of the population, because they were able to perform a cardiopulmonary exercise test and able to walk for 5 minutes without interruption. Therefore, we believe that the difference in relative aerobic load that we found is representative of the difference between populations.

Finally, we need to acknowledge that this study was performed in people in the subacute phase after stroke. It would be of interest to see how our results can be generalized to individuals in chronic stages after stroke.

### Conclusion

Individuals after stroke experience a high relative aerobic load during cyclic daily-life activities, despite adopting a lower movement pace than peers with no impairments. Possibly, individuals after stroke limit their movement pace as a strategy to act at sustainable relative aerobic load levels. High relative aerobic load, concomitant fatigue, and a low movement pace can limit daily-life functioning. Hence, in addition to improving neuromuscular control through neurorehabilitation, improving aerobic capacity through structured aerobic training should be further investigated in the rehabilitation program as a potential intervention to improve mobility and functioning after stroke.

## Supplementary Material

PTJ-2021-0364_R2_Supplementary_Appendices_pzad005

## Data Availability

Raw data were generated at Heliomare, Wijk aan Zee, the Netherlands. Derived data supporting the findings of this study are available from the corresponding author on request.

## Clinical Trial Registration

This study was registered in the Dutch Trial Registry (NL-64431.029.18).
