# Supplementary material for: Relative Aerobic Load of Daily Activities After Stroke
Source: Phys Ther. 2023 Jan 16;103(3):pzad005. doi: 10.1093/ptj/pzad005 (PMC10071588; doi:10.1093/ptj/pzad005)
Supplement: PTJ-2021-0364_R2_Supplementary_Appendices_pzad005 [file ptj-2021-0364_r2_supplementary_appendices_pzad005.pdf]

# Appendix I - Setup

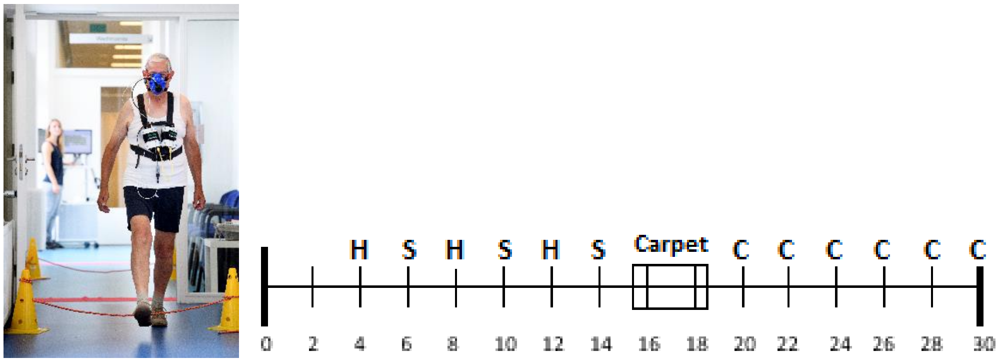


***Setup of the 30 meters obstacle course. walking with obstacles of hurdles (rope between two cones, 10 cm high), flat slats (15-cm large), cross a foam carpet (1-cm thick) and walk around cones (2-m slalom). H, hurdles; S, slats; C, cones.***

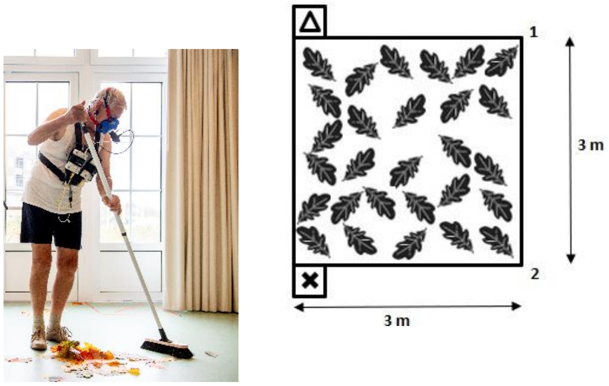


***Setup of the sweeping trial. The participant start from 1 to X, thereon from 2 to Δ.New leaves were added to the square after the participant swept the square clean.***

# Appendix II - Trial in- and exclusion

| | | FAC 3 (N=14) | FAC 4 (N=25) | FAC 5 (N=40) | Able-bodied (N=22) |
|---|---|---|---|---|---|
| **Walking** | Complete | 14 | 25 | 38 | 22 |
| | Not performed | 0 | 0 | 1<br>Time constraint | 0 |
| | Exclusion | 0 | 0 | 1<br>No steady state | 0 |
| **Obstacle walking** | Complete | 8 | 22 | 38 | 22 |
| | Not performed | 5<br>Not possible due to function (5) | 2<br>Not possible due to function (2) | 0 | 0 |
| | Exclusion | 1<br>Performed without obstacles | 1<br>No steady state | 2<br>No steady state(2) | 0 |
| **Stair ambulation** | Complete | 10 | 23 | 34 | 21 |
| | Not performed | 3<br>Balance (2),muscle weakness(1) | 1<br>Unknown | 1<br>Physician' advice | 0 |
| | Exclusion | 1<br>Stopped prematurely | 1<br>No steady state | 5<br>No steady state (2), stopped prematurely(3) | 1<br>No steady state |
| **Sweeping** | Complete | 5 | 23 | 38 | 22 |
| | Not performed | 9<br>Balance problems(9) | 2<br>Fatigue (1)<br>Unknown(1) | 1<br>Unknown | 0 |
| | Exclusion | 0 | 0 | 1<br>Measurement error | 0 |
| **Cycling** | Complete | 14 | 22 | 39 | 22 |
| | Not performed | 0 | 2<br>Knee injury (1)<br>fatigue(1) | 0 | 0 |
| | Exclusion | 0 | 1<br>Stopped prematurely | 1<br>Unknown | 0 |
| **Total** | **Complete** | **51/70 (72.6%)** | **115/125 (92%)** | **188/200 (94%)** | **218/220 (99.1%)** |
| | **Not performed** | **17/70 (24.3%)** | **7/125 (5.6%)** | **3/200 (1.5%)** | **0/110 (0%)** |
| | **Exclusion** | **2/70 (2.9%)** | **3/125 (2.4%)** | **9/200( 4.5%)** | **2/220 (0.9%)** |
